# Biodegradable nano-reinforced packaging with improved functionality to extend the freshness and longevity of Plums *Oemleria cerasiformis*

**DOI:** 10.1038/s41598-023-41640-1

**Published:** 2023-09-04

**Authors:** Manpreet Kaur Mann, Balwinder Singh Sooch

**Affiliations:** Enzyme Biotechnology Laboratory, Department of Biotechnology, Punjabi University, Patiala, 147002 India

**Keywords:** Environmental sciences, Nanoscience and technology

## Abstract

Food packaging reinforced with Zn-doped TiO_2_ nanoparticles with enhanced prerequisite film-forming and biodegradable traits was prepared to augment fresh food storage. Pure and tailored metal (Zinc, Copper, and Selenium) doped TiO_2_ nanoparticles were synthesized and analyzed through multiple characterization techniques (optical spectra, XRD patterns (X-Ray Diffraction), Dynamic Light Scattering, and Scanning Electron Microscopy). The synthesized nanoparticles were tested for their Minimum Inhibitory Concentrations, antimicrobial potential against common lethal food pathogens, and cytotoxicity. Compared to Cu- and Se-doped nanoparticles, Zn-doped TiO_2_ nanoparticles displayed the most potent antimicrobial activity with insignificant cytotoxicity and were incorporated into the food packaging materials. The developed nano-reinforced food packaging efficaciously augmented the freshness of plums (*Oemleria cerasiformis*) for 16 days (42 ± 2 °C). The physicomechanical characterization of the nano-reinforced packaging establishes its utility in food packaging applications. The developed biodegradable packaging undergoes complete decomposition within 12 days of storage in natural soil.

## Introduction

Food spoilage from improper food storage (packaging materials/techniques) leads to losses in the food industry. The FAO (Food and Agriculture Organization of the United Nations) conveyed that more than 1.3 billion metric tons of usable food are lost or squandered every year mainly due to poor post-harvest practices, packaging, transport conveniences, and at the consumer level^1^. However, it is mandatory to control food wastage to combat food crises and address the emerging challenge of feeding the growing population. The chief origin of food wastage is microbial spoilage that makes the food unfit for human consumption^2^. Active packages have invaded the food packaging commerce for several ages, which includes the amalgamation of certain functional materials into food packages. Functionalized food packaging, including biodegradable materials, alters the internal atmosphere of the packaged food, providing improved traits and safety for human consumption. Hence, there is an urgent need to develop and use biodegradable active natural resources as a sole source of food packaging materials^3^. Bio-based polymers produced from natural carbohydrates and proteins have emerged as excellent barriers to oxygen due to their compact hydrogen-bonded network chains. Gelatin, a protein-based biopolymer obtained from fish and widely used for edible film formation, has shown limited utility in food packaging due to its lower moisture barrier attributes^4^. Pectin, another film-forming polymer that is water-soluble and highly hygroscopic, is well recognized in the food and pharmacological industries as an encapsulating material due to its excellent thickening, gelling, and stabilizing capabilities^5^. Since both types of biopolymer materials possess several deficiencies related to poor water barrier, blending gelatin with pectin may offer benefits in terms of its improved physicomechanical aspects and moisture barrier rather than preparing films from only gelatin or pectin^6^.

Nanotechnology has several practical implications in food production, processing, storage, and distribution. Several metals and metal oxide nanoparticles in the form of inorganic nanoparticles, namely silver, zinc oxide, iron, carbon, silicon dioxide, titanium oxides, and magnesium oxide, have practical implications as antimicrobial-based food additives^7^. Amongst the inorganic nanoparticles, titanium and zinc oxide have emerged as efficient additives to food packaging that enhance storage functionality due to their Generally Recognized Safe (GRAS) status. TiO_2_ was allowed as a food additive in bulk quantities (E171). TiO_2_ is a naturally occurring material widely utilized as a brightening and whitening food additive pigment in the confectionery, baking industry, and chewing gums with no maximum level specified^8–10^. Titanium dioxide is a stable, non-toxic, economical, and GRAS material owing to its photocatalytic nature and has been regarded as an intriguing antibacterial agent^11^. The chitosan-based film reinforced with nanosized TiO_2_ nanoparticles and red apple pomace extract was used to monitor the freshness of salmon fillets. The reinforced film showed remarkable antioxidant and antibacterial properties^12^. TiO_2_-reinforced chitosan-based nanocomposite film was synthesized using tannic acid as a crosslinking agent with improved mechanical properties. The prepared films possessed better thermal stability with reduced levels of swelling and water vapour permeability. The developed films showed potent antioxidant activity and had great potential to be used as active food packaging^13^. A TiO_2_-reinforced active and biodegradable food packaging film was prepared from poly (butylene adipate-co-terephthalate) (PBAT) and poly (lactic acid) (PLA) that extended the shelf life of grapes^14^.

Using two or more potent nanoparticles provides a synergistic effect called doped nanoparticles, thus demonstrating effective antimicrobial potential compared to a single metal nanoparticle. The current study presents the impact of TiO_2_ nanoparticles and their food-grade dopants, such as zinc, copper, and selenium metals, on the proficiency of the gelatin-pectin blended food packaging films. The physicochemical properties of the amalgamated nanoparticles and prepared nano-reinforced food packaging films were analyzed and compared with neat gelatin-pectin films. To our understanding, this has been the first report implying the functionality of the nano-reinforced gelatin-pectin active food packaging material for augmenting the quality of fresh foodstuffs. The efficiency of the prepared nano-reinforced packaging was evaluated to extend the shelf-life of plums while preserving their paramount quality.

## Experimental

### Chemicals

The chemicals employed in the present work were of analytical grade with high purity procured from Hi-media, Merck, Qualigens Fine chemicals, and Sisco Research Labs Pvt. Ltd. Deep red plums (*Oemleria cerasiformis*) were procured from the local market near Punjabi University, Patiala (India) campus.

### Synthesis procedure for pure TiO_2_ and doped TiO_2_ nanoparticles

Pure TiO_2_ nanoparticles were synthesized via a simple facile hydrolysis technique^15^. Similarly, Zn, Cu, and Se-doped TiO_2_ nanoparticles were synthesized following the same protocols with some modifications. Initially, zinc nitrate, copper nitrate, and selenium metal (0.05 M) were added to stock TX-100 solution under high stirring speed for about 1 h to obtain Zn-doped, Cu-doped, and Se-doped TiO_2_ nanoparticles, respectively, later following the same procedure as mentioned above for pure TiO_2_ nanoparticles.

### Physico-chemical properties of the synthesized metal nanoparticles

The synthesized nanoparticles were visually observed for their morphology and UV–Vis spectrum (200-800 nm) using a UV–Vis spectrophotometer (Shimadzu, UV-1800, Switzerland). The size of the synthesized nanoparticles was measured using Malvern Panalytical Zetasizer Nano ZSP operated at 25 °C DLS (dynamic light scattering). Samples were prepared by dissolving 0.2 g of nanoparticles powder in 100 mL of distilled water (filtered using a PES syringe filter with a pore size of 0.22 µm). The dissolved nanoparticles suspension was sonicated for 50 min with a pulse of 25 s on and 5 s off. The sonicated samples were filtered with a PES syringe filter to remove larger particles. The samples were then subjected to DLS measurements. XRD patterns of the synthesized pure and doped TiO_2_ nanoparticles were examined by X'PERT PRO PAN analytical instrumental operated at a current of 40 mA (Cu K_α_ radiation, K_α_ = 1.54060 Å) in the transmission mode at 45 kV voltage at a scan rate of 0.0080° and a scan step time of 10.16 s. The average size of the synthesized nanoparticles was identified using Scherrer’s equation.

### Minimum inhibitory concentrations (MIC) and antibacterial potential of the synthesized nanoparticles

The Minimum Inhibitory Concentration (MIC) is the lowermost concentration of the antimicrobial agent, which completely obstructs the progression (99%) of the test microorganism. MIC for the synthesized pure and doped TiO_2_ nanoparticles was evaluated by Duffy et al.^16^ with some alterations. The MIC was tested against Gram-positive (*S. aureus* and *B. pumilus*) and Gram-negative (*E. coli* and *P. oleovorans*) in tubes or microdilution wells determined by the visual turbidity in the tubes^16^. The method involves preparing dilutions of the synthesized nanoparticles as antimicrobial agents in the 5, 10, 25, 50, and 100 µg/mL concentrations in nutrient broth with 2 mL of minimum volume. Next, each tube was inoculated with test inoculum (1 × 10^6^ cells/mL) adjusted to 0.5 McFarland scale. The inoculated tubes were further incubated at 37 °C for 24 h, and the microbial growth was further scrutinized by evaluating the optical density (OD) at 620 nm. Negative control was taken as broth and nanoparticles, whereas a positive control was taken as nutrient broth and bacterial suspension.

The antibacterial potential of the prepared nanoparticles was tested against two Gram-positive bacteria, namely *S. aureus* and *B. pumilus* strains, and two Gram-negative bacterial cells, *E. coli* and *P. oleovorans,* i.e., via well diffusion approach as suggested by Sooch and Mann^17^. The respective bacterial inoculums (5μL) consisting of 3.1 × 10^6^ cfu/mL of bacterial suspension prepared by McFarland turbidity standard (0.5) were inoculated in plates containing solidified nutrient agar and spread evenly with the help of a sterilized spreader. After that, a well of 6 mm diameter was bored in the inoculated solidified agar plates using a sterilized steel borer. Dissimilar concentrations (25–200 µg/mL) of each type of synthesized nanoparticles (pure and doped TiO_2_ nanoparticles) were loaded into the wells made in agar plates and incubated for 24 h at 37 °C to inspect their antimicrobial susceptibility. Hi-media Antibiotic Zone Scale TM-C measured the zone of inhibition formed around the wells.

### Minimum inhibitory concentrations (MIC) and antifungal potential of the synthesized nanoparticles

MIC for the synthesized nanoparticles was assessed through Duffy et al.^16^ modified approach^16^. The MIC was tested against four fungal strains: *Botrytis cineria* MTCC 2104, *Candida albicans* MTCC 3017*, Fusarium oxysporum* MTCC 1755, *and Penicillium expansum* MTCC 4485 in tubes or microdilution wells determined by the visual turbidity in the tubes. The method involves preparing dilutions of the synthesized nanoparticles in the 5, 10, 25, 50, and 100 µg/mL concentrations in a nutrient broth of 2 mL of minimum volume. Afterward, each tube was inoculated with test inoculum (1 × 10^6^ spores/mL) adjusted to a 0.5 McFarland scale. A negative control was taken as broth and nanoparticles, whereas a positive control was taken as nutrient broth and fungal suspension. The inoculated tubes were further incubated at 25 °C for 36 h. The microbial growth was scrutinized by assessing the optical density (OD) at 620 nm.

The antifungal efficacy of the synthesized nanoparticles was investigated using the well-diffusion method against *Botrytis cineria* MTCC 2104, *Candida albicans* MTCC 3017*, Fusarium oxysporum* MTCC 1755 *and Penicillium expansum* MTCC 4485. The respective media for each culture was poured into sterilized petri plates, to which separate spore suspensions consisting of 1 × 10^7^ spores/mL prepared using McFarland turbidity standard (0.5) were seeded with the help of a sterilized glass spreader. A well (6 mm diameter) was bored with the help of a sterilized stainless steel borer. Variable concentrations (25–200 µg/mL) of nanoparticles synthesized were poured into the wells of separate Petri plates. The inoculated plates were further incubated at 25 °C for 2–4 days until a clear zone of inhibition was observed surrounding the wells. The clear zone of inhibition around the wells was detected using Hi-media Antibiotic Zone ScaleTM-C.

### Cytotoxicity of the synthesized nanoparticles

The viability of spleenocytes after acquaintance with synthesized nanoparticles was evaluated via MTT colorimetric test^17^. In this assay, 2.4 × 10^6^ cells/mL were placed in 96-well plates and exposed to different concentrations of nanoparticles (5, 10, 25, 50, 100, 150, 200 µg/mL) for 72 h. After complete exposure of each well, the old medium was changed out for a fresh one that contained the MTT solution (equal to 10% of culture volume). The cells were further incubated for 4 h at 37 °C in the presence of CO_2_ (5%) till the purple-coloured formazan product got solubilized in DMSO, and the absorbance 570 nm was measured by using a Thermo Scientific Microplate Photometer.

### FE-SEM analysis

The nanoparticles with the utmost antimicrobial and lower cytotoxic response were explored for their morphological characterizations through Cold Field Emission Scanning Electron Microscope (Hitachi, SU 8010). The powdered nanoparticle samples were used directly for SEM analysis.

### Reinforcement of the synthesized nanoparticles in active antimicrobial food packages

The solution casting method fabricated the nano-reinforced active food package prepared from gelatin and pectin. The gelatin and pectin solutions were prepared by mixing gelatin (8 g) and pectin (8 g) separately in 100 mL distilled water and kept for 30 min at room temperature, and then glycerol (25%, w/w total gelatin) was added and stirred at 55 °C for 30 min. The most competent antimicrobial nanoparticles were added under stirring conditions for 30 min (room temperature). Similarly, the control films were prepared without adding nanoparticles. The film mixture was then cast on a leveled tray and kept for drying for 48 h. The films prepared were peeled and kept for further use.

### Analysis of the prepared food packaging films

#### Physico-mechanical characterizations of the prepared films

The prepared films were stored in a desiccator, and small strips of 4*4 cm were cut and used for further characterization. The prepared films were measured for their thickness using a digital vernier calliper (Mitutoyo Corporation, Japan, model-CD-12), having the least count of 0.01 mm^17^. The film thickness was determined by taking the random average of ten values. To determine the moisture content of the prepared films, film pieces were dried for 24 h at 105 °C in a hot air oven, and the dry weight (*w*_1_) was recorded^18^. The experiment was performed in triplicates, and the moisture content (*mc*) was measured from the weight loss using the below equation:

$$mc = \frac{{w_{0} - w_{1} }}{{w_{0} }} \times 100.$$

To determine the prepared films’ water solubility index and swelling behaviours, the film pieces were dried at 105 °C for 12 h, and their weight was measured as *m*_1_. The dried film sample was soaked in 50 mL distilled water for 24 h under steady shaking conditions, and the weight of the sample was taken again as *m*_2_ after incubation. Finally, the film sample was dried at 105 °C for 12 h and weighed as *m*_3_.

The swelling index (*w*_*sb*_ %) was calculated as follows:

$$w_{sb = } \frac{{m_{2} - m_{1} }}{{m_{1} }} \times 100.$$

Water solubility behaviour (*w*_*ws*_ %) was calculated as follows:

$$w_{ws = } \frac{{m_{1} - m_{3} }}{{m_{1} }} \times 100.$$

The prepared film samples' water vapour permeability was performed using an aluminum cup with 2.5 cm × 5.4 cm (height × width) by the method suggested by Farahnaky et al.^19^. The aluminium cup was filled up to the brim with dry silica gel, and the mouth of the cup was sealed with a film sample. The cup and the film were placed in a desiccator (with super-saturated sodium chloride solution with 75% relative humidity at 25 °C). The assembly was then weighed regularly 8 times after every hour interval. *WVP* was calculated from the following expression:

$$WVP = \frac{WVTR}{{\Delta P}} \times L$$

where *L* is the film thickness, *WVTR* is the water vapour transmission rate (g m^−2^ h^−1^) measured through a film, calculated from the slope of the straight line divided by the exposed film area (m^2^). *ΔP* is the difference in partial water vapor pressure (Pa) across both sides of the film, and measurements were performed in triplicates.

Mechanical properties such as Tensile strength (TS) and Elongation at break (EB) of the prepared films were performed using a texture analyzer (TA.XT plus, Stable Micro Systems Ltd, UK) by following standard protocols^20^. Each film strip (2.54 × 6.0 cm) was set with an initial grip separation of 40 mm and a mechanical cross-head speed of 1 mm/s. The individual film sample was verified in triplicates with average values. The Tensile strength (*TS*), Elongation at break (%*EAB*) of the film samples were measured through the following expressions:

$$TS\left( {{\text{MPa}}} \right) = \frac{{F_{max} }}{A}$$

where *F*_*max*_ = maximum load (N) required to pull the film strip apart and *A* = average cross-sectional area (m^2^) of the sample.

$$EAB = \frac{E}{40} \times 100$$

where E = Elongation of the film (mm) at the moment of break, 40 = Initial grip separation of the analyzer.

The bursting strength of the film samples was also performed using the TA.XT plus, Stable Micro Systems Ltd, UK.

#### Contact angle measurements

The hydrophobic characteristics of the prepared film samples were estimated via the sessile drop approach^17^. A petriplate sized film samples were used and positioned on a flat surface. A small drop of millipore water was placed on the surface of the films with the help of a micropipette, and images were taken with a digital camera. The further contact angle was measured in Image J software.

#### Color and opacity measurements

Spectrocolorimeter (X-rite, RM 200, QC, Grand Rapids, Michigan, USA) was employed to calculate the color of the prepared films. Film samples were measured on a standard white plate with L* = 90.7, a* = 6.3, b* = -16.3, indicating the lightness as *L* and chromaticity parameters *a* as red to green and *b* as yellow to blue. All the colors can be expressed by *L* {0 (black)–100 (white)}, a values {negative (greenness)-positive (redness)} and b values {negative (blueness) − positive (yellowness)}. All the measurements were performed in triplicate, and *ΔE* as total color difference, *WI* as whiteness index, and *YI* as yellowness index were determined from the following expressions:

$$\Delta E = \sqrt {\left( {L^{*} - L} \right)^{2} + \left( {a^{*} - a} \right)^{2} + \left( {b^{*} - b} \right)^{2} }$$

$$WI = 100 - \sqrt {\left( {100 - L} \right)^{2} + a^{2} + b^{2} }$$

$$YI = \frac{142.86 \times b}{L}.$$

The UV–visible absorbance of the prepared films was measured via a UV–VIS spectrophotometer^21^, and the opacity of the films was measured via the following expression:

$$Opacity = \frac{{A_{600} }}{x}$$

*A*_600_ is the absorbance at 600 nm wavelength, and *x* is film thickness (mm).

### Antimicrobial potential of the prepared nanopacks

The antibacterial potential of the prepared films was evaluated via the agar disc diffusion method^22^. Four food-borne pathogenic bacteria were tested for susceptibility towards the films, namely *B. pumilus*, *S. aureus*, *P. oleovorans,* and *E. coli* MTCC 1687. Bacterial Suspensions following McFarland turbidity standard (0.5) containing (3.1 × 10^6^ CFU/mL) colonies of each tested strain were spread on the surface of the prepared sterilized agar plates. The film samples cut in a round disc of 12 mm diameter were placed on the surface of the pre-prepared agar plates and incubated for 24 h at 37 °C. After incubation, the inhibition zone diameter around the mounted film samples was measured in triplicates using a standard Hi-media zone scale.

Antifungal susceptibility of the prepared films against *B. cineria*, *C. albicans, F. oxysporum, and P. expansum* was examined by the disk diffusion method, as suggested by Salaberria et al.^23^. An aliquot of approximately 1 × 10^7^ (CFU/mL) colonies of each tested strain was prepared in Tween 80 (0.01 v/v) using McFarland turbidity standard (0.5) and was cultured on the pre-prepared potato dextrose agar (PDA)^23^. A thin pellet of each film (measuring 12 mm diameter) was mounted on cultured plates and incubated for 72 h at 25 °C. After incubation, the inhibition zone surrounding the film pellet was measured via a standard Hi-media zone scale.

### Biodegradation examination of the prepared films

The biodegradability of the prepared nano-reinforced packaging into natural soil was measured via indoor soil burial degradation protocol^24^ with some alterations. Natural soil was collected from the University campus and placed onto small mud pots to be used as a degradation setup. Each film sample (2 × 3 cm^2^) was dried for 24 h in a hot air oven at 70º C, and the primary weight of the films was measured as *m*_0_. After that, dried film pieces were kept in a metal (aluminium) web and placed 5 cm deep into collected natural soil in mud pots. The pots were watered regularly to retain their moisture level. The degradation evaluation of the film was assessed on the 5th, 7th, 9th, 16th, 32nd, 62nd day and 92nd day in terms of loss of film weight (%) by removing the films out from the aluminium web, washed under running tap water and finally drying for 24 h under hot air oven. The final weight of the corresponding films was recorded as *m*_*1,*_ and the weight loss (%) of the films was calculated via the below expression:

$$wl\left( \% \right) = \frac{{m_{0} - m_{1} }}{{m_{0} }} \times 100.$$

### Evaluation of the prepared films for the shelf life enhancement

The proficiency of prepared nano-reinforced packaging for enhancing the shelf life of plum (*Oemleria cerasiformis*) was measured by taking fresh and firm plums of the same size, shape, and colour. “The use of plants/plant parts (Plum) in the present study complies with international, national, and/or institutional guidelines”. Fresh Plums were initially washed and sterilized with sodium hypochlorite solution. The plums were again washed under running water and, finally, air dried. A layer of each type of the prepared film was wrapped around a set of plums and sealed. One set of the plums was covered with plastic (polyethylene) cling film as a negative control, another set was taken as a positive control film (without reinforcing nanoparticles), and another was kept untreated. Finally, the fourth set of plums was covered in a prepared nano-reinforced packaging. The wrapped plums were then kept inside on the workroom shelf at approximately 42 ± 2 °C and examined regularly for their freshness for several days.

### Microbial load investigation of the films evaluated as food packaging material

The TBC (total bacterial count) and TYC (total yeast and fungi count) of the film samples were assessed by taking a piece of the film (5–10 g) removed from the stored plums and aseptically transferred into sterile 0.85% (w/v) NaCl solution and homogenized. All the evaluations were executed in triplicates and expressed as CFU/mL. Serial decimal dilutions were prepared in sterile saline, and 5 µL of the aliquot was spread onto the prepared nutrient agar and potato dextrose agar plates by incubating them separately at 37 °C and 25 °C for 48 h, respectively.

### Statistical evaluation

All the data obtained were expressed as means (triplicates) ± standard error with Tukey’s hinges using Origin Pro 2022 (OriginLab Corporation) software. Statistical differences were considered at a significance level of *p* ≤ 0.05.

## Results and discussion

Pure and doped TiO_2_ nanoparticles were synthesized through a basic hydrolysis approach. It has been observed visually that pure, Cu-doped, and Zn-doped TiO_2_ nanoparticles were crystalline milky white coloured powders. In contrast, Se-doped TiO_2_ nanoparticles appeared as greyish-white powders. Keswani et al. also reported the white colour of titanium dioxide nanoparticles^15^.

**Figure 1:**
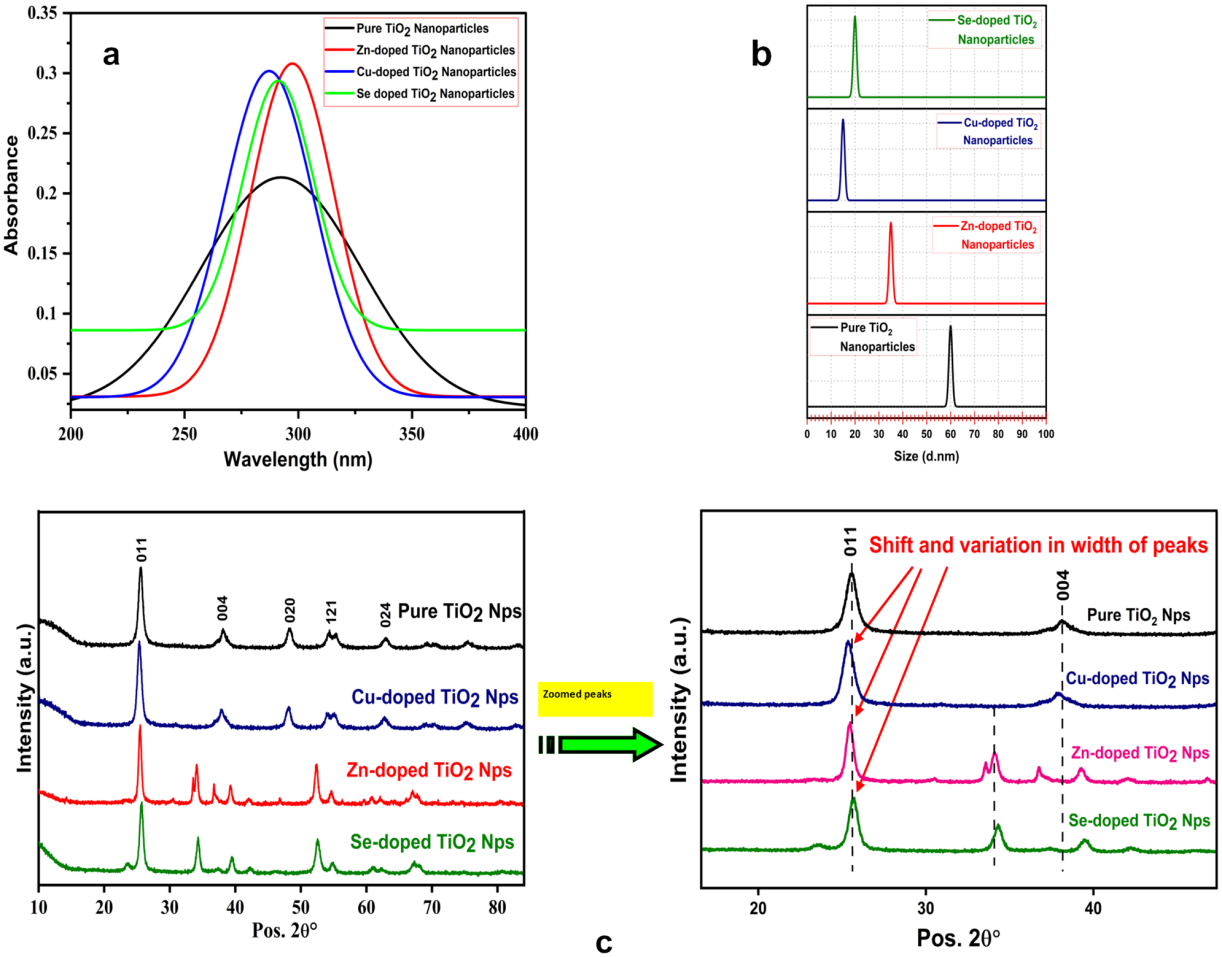
(**a**) UV–visible spectral analyses of pure and metal-doped TiO_2_ nanoparticles; (**b**) DLS analysis of pure and metal-doped TiO_2_ nanoparticles; (**c**) X-Ray Diffraction patterns of pure and metal-doped TiO_2_ nanoparticles.

Figure 1a presents the UV–Vis spectrum of the synthesized nanoparticles, which reveals that pure TiO_2_ nanoparticles show a strong absorption peak at 280 nm. In contrast, the absorption spectrum in the range of 290–320 nm might be due to the formation of new species of TiO_2_ due to the diverse incorporation of dopants. In addition, metal dopants (Zn, Cu, and Se) were also responsible for the formation of defects inside the structure of pure TiO_2_ nanoparticles, resulting in the shift of absorption edge towards a higher wavelength region (redshift) for Zn- and Se-doped and shift of absorption edge towards a lower wavelength region (blueshift) for Cu-doped, which is characteristic for TiO_2_ nanoparticles after incorporation of dopants. A similar redshift has also been identified by Wang et al.^25^ from pure to Cu-doped TiO_2_ nanoparticles and also by Khairy and Zakaria^26^ from pure to Zn-doped TiO_2_ nanoparticles, respectively.

The sizing characterization of the synthesized pure TiO_2_ nanoparticles revealed a size range of 59.90 ± 0.01 nm. The doped TiO_2_ nanoparticles exhibited different size ranges of 35.0 ± 0.02 nm, 15.10 ± 0.02 nm, and 20.10 ± 0.03 nm for Zn-doped, Cu-doped, and Se-doped TiO_2_ nanoparticles, respectively (Fig. 1b). The stacked plots for pure and doped TiO_2_ nanoparticles showed peak shifting from their bulk counterparts. This is generally attributed to incorporating dopants into the lattice structures of the TiO_2_ nanoparticles. The characteristic peaks for the doped TiO_2_ nanoparticles are mainly due to the incorporation of dopants (Zn, Cu, Se) into the structure of TiO_2_ nanoparticles, thereby causing shrinking of the lattice parameters that were eventually reducing the size of doped nanoparticles. The zeta-sizing analysis of the synthesized nanoparticles aligned with the grain size obtained through XRD analysis. The DLS analysis further reveals that the synthesized nanoparticles were mono-disperse in nature without any aggregation in the medium. The hydrodynamic size of 317 nm for pure and 269–289 nm for Zn-doped TiO_2_ nanoparticles has been investigated by Ahamed et al.^27^.

XRD patterns of the synthesized nanoparticles are shown in Fig. 1c. The sharp and strong diffraction peaks denote the pureness of the formed product. The well-defined diffraction peaks with 2θ are at about 25°, 37°, 48°, 55°, and 62°, which were assigned to (011), (004), (020), (121) and (024), crystal plane, respectively. The identified diffraction peaks were indexed, suggesting a tetragonal phase for pure TiO_2_ nanoparticles, with calculated lattice parameters, a = 3.8 Å, C = 9.5 Å, which confirms the anatase phases of the TiO_2_ (JCPDS FILE No. 21–1272). It is well known that the anatase form was the most stable form of TiO_2_. Cu-doped TiO_2_ nanoparticles possess lattice parameters such as a = 3.8 Å and c = 9.5 Å with a tetragonal crystal system. The lattice parameters calculated for Zn-doped TiO_2_ are a = 3.2 Å and c = 5.1 Å with a hexagonal crystal system. In addition, the lattice parameters for Se-doped TiO_2_ nanoparticles were calculated as a = 4.6 Å, c = 2.9 Å, with a Tetragonal crystal system. The pure and Cu-doped TiO_2_ nanoparticles possess the same values for lattice spaces, whereas Zn-doped and Se-doped TiO_2_ nanoparticles have variations in their lattice parameters from the host. Moreover, no additional peaks have been observed for the cu dopant, indicating that the copper species has occupied the substitution sites in the titanium dioxide matrix without distorting the host lattices. Yadav et al. also reported no deviation of the peak position with varying dopant concentrations for Cu-doped TiO_2_ nanoparticles^28^. A single additional peak has been observed for Zn-doped TiO_2_ and Se-doped TiO_2_ towards lower 2θ values. This is, however, due to the change in the ionic radii of the dopants. None of the additional peaks or traces for secondary phases or impurities has been experienced. The most intense peak (identified at 2θ ~ 25) determines the average crystallite size of all the synthesized nanoparticles from the FWHM of the anatase (011) reflection plane using Scherrer’s formula,

$$D = \frac{\kappa \lambda }{{\beta cos\theta }}$$

where D is the crystallite size, k is a constant (0.9), and λ is the wavelength of the X-ray diffraction. β is the FWHM, and θ is the angle of diffraction.

The calculated crystallite size of the nanoparticles reveals that the synthesized samples had miniature grain size with an average crystallite size of 55.6 nm for the pure TiO_2_ nanoparticles and 31.5 nm, 13.7 nm, and 15.7 nm for Zn-doped, Cu-doped, and Se-doped TiO_2_ nanoparticles, respectively. It has been further demonstrated from the calculated average size of various dopants that Cu-doped TiO_2_ has been found to have the smallest nanosize as compared to all other synthesized nanoparticles. Also, it has been depicted from the above analysis that the size has been critically reduced from the pure form for every metal dopant. It has also been observed that the size of pure TiO_2_ nanoparticles has decreased from 38.4 to 29.2 nm for Zn-doped TiO_2_ nanoparticles, demonstrating that doping reduces the size of pure TiO_2_^29^. Ramimoghadam et al. also observed a diffraction pattern for TiO_2_ nanoparticles^30^.

The synthesized nanoparticles were evaluated for their MIC and antibacterial potential against Gram-positive bacteria (*B. pumilus* and *S. aureus* strains) and two Gram-negative bacteria (*P. oleovorans* and *E. coli*). Different concentrations (25–200 µg/mL) of each synthesized nanoparticles (Pure, Zn-doped, Cu-doped, Se-doped TiO_2_ nanoparticles) were tested against all the selected bacterial strains.

The inhibitory assay conducted for pure and doped TiO_2_ nanoparticles has shown MIC values of 25 µg/mL for pure TiO_2_ nanoparticles against the Gram-positive bacterial strains (*S. aureus* and *B. pumilus*), as shown in Table 1. Interestingly, the titanium tetrachloride (precursor) used initially to prepare TiO_2_ nanoparticles has shown no inhibition effect against the tested Gram-positive bacterial strains (*B. pumilus* and *S. aureus*). The MIC values were also determined for the synthesized pure and doped TiO_2_ nanoparticles against the Gram-negative bacterial strains (*P. oleovorans* and *E. coli*), as shown in Table 1. The MIC values for pure TiO_2_ nanoparticles are 25 µg/mL against *P. oleovorans* and *E. coli*. The Zn-doped, Cu-doped, and Se-doped TiO_2_ nanoparticles have 10 µg/mL MIC values against *E. coli* and 25 µg/mL against *P. oleovorans*. The doped TiO_2_ nanoparticles have more inhibitory effects against *E. coli* than *P. oleovorans* at the minimum concentration. The titanium tetrachloride has no inhibitory effect against the Gram-negative bacterial strains at any tested concentrations. The minimum inhibitory assay determined for the synthesized pure and doped ZnO, CuO, SeO_2_, and TiO_2_ nanoparticles against Gram-positive and Gram-negative bacterial cells revealed that the synthesized doped nanoparticles showed greater inhibition towards the tested Gram-negative bacterial cells as compared to the Gram-positive bacterial cells. The stronger antimicrobial behaviour of the doped nanoparticles is majorly attributed to the bimetallic effect of two metals that synergistically enhances the inhibition traits against the tested microbes. The DLS and XRD analysis determined that the doped nanoparticles had attained a smaller size than their pure form. The smaller the size, the greater the inhibition of the microbes, as the small particles may get attached to the bacterial cell walls, thereby causing leakage of their cellular components, leading to the death of the microbes.

**Table 1:** Minimum inhibitory concentration (µg/mL) of pure and metal-doped titanium dioxide nanoparticles against gram-positive and gram-negative bacterial strains.

Conc. (µg/mL)	Bacterial strains (gram-positive)	Minimum inhibitory concentration (µg/mL)				
		Pure and metal-doped TiO_2_ nanoparticles				
		Pure TiO_2_ nanoparticles	Cu-doped TiO_2_ nanoparticles	Se-doped TiO_2_ nanoparticles	Ti-doped TiO_2_ nanoparticles	Titanium tetrachloride (precursor)
5	*B. pumilus*	+	+	+	+	+
	*S. aureus*	+	+	+	+	+
	*P. oleovorans*	+	+	+	+	−
	*E. coli*	+	+	+	+	−
10	*B. pumilus*	+	+	+	+	+
	*S. aureus*	+	+	+	+	+
	*P. oleovorans*	+	+	+	+	−
	*E. coli*	+	−	−	−	−
25	*B. pumilus*	−	−	−	−	+
	*S. aureus*	−	−	−	−	+
	*P. oleovorans*	−	−	−	−	−
	*E. coli*	−	−	−	−	−
50	*B. pumilus*	−	−	−	−	+
	*S. aureus*	−	−	−	−	+
	*P. oleovorans*	−	−	−	−	−
	*E. coli*	−	−	−	−	−
100	*B. pumilus*	−	−	−	−	+
	*S. aureus*	−	−	−	−	+
	*P. oleovorans*	−	−	−	−	−
	*E. coli*	−	−	−	−	−
200	*B. pumilus*	−	−	−	−	+
	*S. aureus*	−	−	−	−	+
	*P. oleovorans*	−	−	−	−	−
	*E. coli*	−	−	−	−	−

Positive (+): Signifying turbidity (growth), Negative (−): Signifying no turbidity (absence of growth).

**Figure 2:**
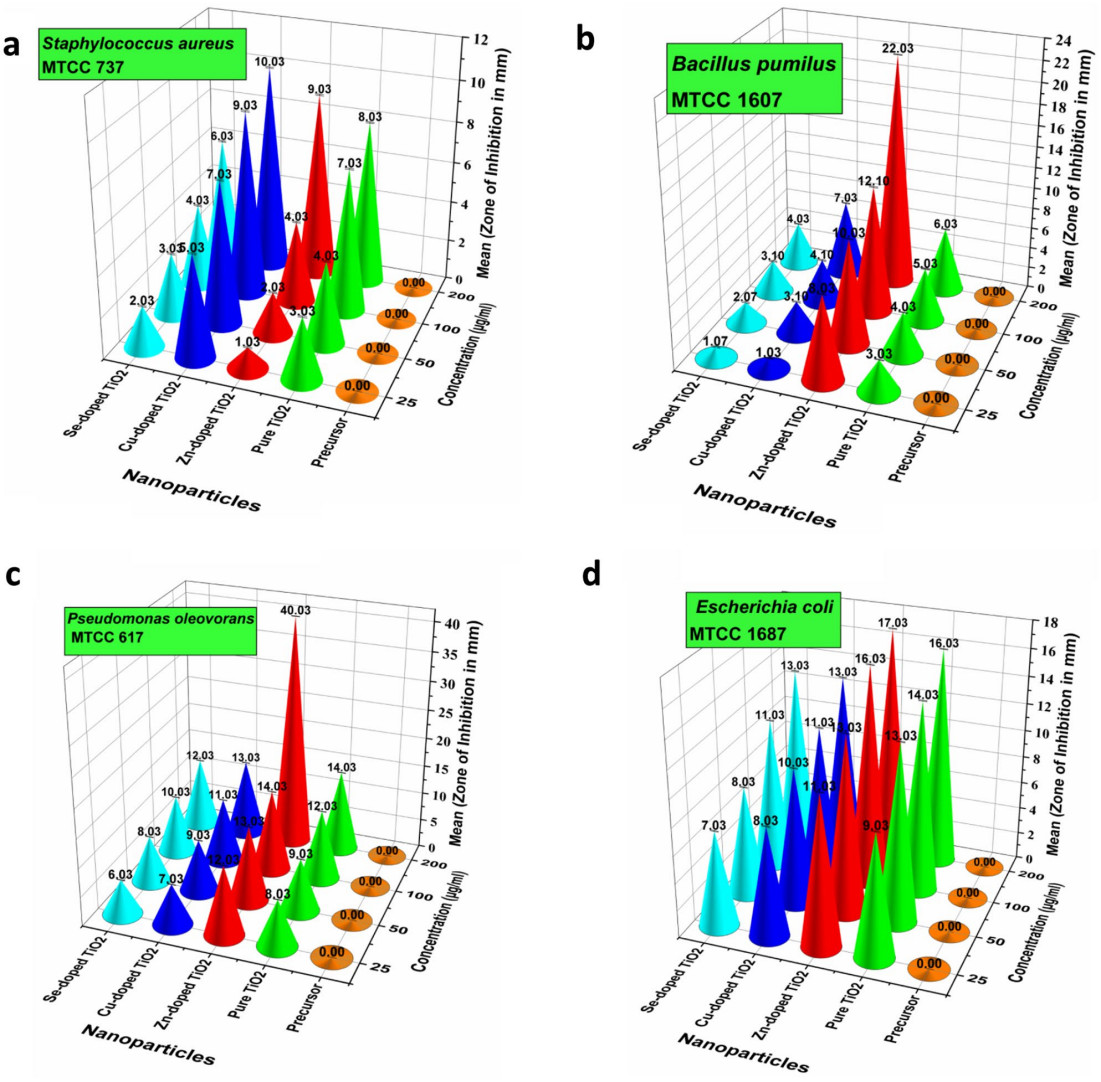
Antibacterial activity of pure and doped TiO_2_ nanoparticles against Gram-positive bacteria (**a**) *S. aureus*; (**b**) *B. pumilus*; Gram-negative bacteria, (**c**) *P. oleovorans*; (**d**) *E. coli*.

It has been depicted from the antimicrobial activity results that Zn-doped TiO_2_ nanoparticles showed the maximum zone of inhibition against *B. pumilus,* as shown in Fig. 2b. The pure, Cu-doped, and Se-doped TiO_2_ nanoparticles also showed significant antimicrobial effects against the same tested bacteria. In the case of another Gram-positive bacteria, i.e., *S. aureus*, the maximum zone of inhibition was shown by Cu-doped TiO_2_ nanoparticles (Fig. 2a). The pure Zn-doped and Se-doped TiO_2_ nanoparticles also showed positive antimicrobial effects against the same tested bacteria. Doped TiO_2_ nanoparticles may have a more substantial antibacterial impact because of the synergistic interaction of the two different metals. In the case of Cu–TiO_2_, the synergistic effects of the concentration of Cu^2+^ ions, production of reactive oxygen species, and binding of Cu^2+^ ions to the bacterial cell surface lead to the inactivation of the proteins present in bacterial cells. Thus, doping significantly impacts the suspension rate of doped TiO_2_ nanoparticles^31^. Doped TiO_2_ nanoparticles demonstrated the best antibacterial effect against *S. aureus* and *E. coli*. Hence, doping nanoparticle is a promising method for creating highly effective antimicrobial medications for bactericidal procedures that do not require antibiotics.

In the case of Gram-negative bacteria, the maximum zone of inhibition is exhibited by Zn-doped TiO_2_ nanoparticles against both the tested strains, i.e., *P. oleovorans* and *E. coli,* as shown in Fig. 2c,d. Compared to pure TiO_2_, the zone of inhibition is explicitly higher, particularly for Zn-doped TiO_2_ nanoparticles against *P. oleovorans* at maximum concentration, i.e., 200 µg/mL. Overall, Zn-doped TiO_2_ nanoparticles exhibited the maximum inhibition zone among the tested Gram-positive and Gram-negative bacterial strains.

TiO_2_ has gained attention due to its antimicrobial potential attained through photocatalytic activity. It has been recognized as a chemically stable, low-cost, non-toxic, readily available material with Generally Recognized as Safe (GRAS) status. Several researchers have explored them as superb antifungal and antibacterial agents against a broad range of Gram-positive and Gram-negative bacterial cells. TiO_2_ nanoparticles possess robust oxidizing command by free radical production, such as hydroxyl and superoxide anion radicals, that inactivates the microbes and sabotages the growth of several microbes, such as *S. aureus* and *E. coli*^11^. Razzaq et al. reported a zone of inhibition of 19 ± 0.14 mm against *Staphylococcus aureus* and 20 ± 0.21 mm against *Escherichia coli* for TiO_2_ nanoparticles at 1 mg/mL concentration^32^.

Notably, based on the observations of the present study, Gram-negative bacteria were found to be more sensitive toward the synthesized nanoparticles than the Gram-positive bacteria strain. Gram-positive bacteria possess thick coatings of peptidoglycan in their cell walls which restricts the entry of nanoparticles into the cells. In contrast, Gram-negative bacterial cells consist of a thin peptidoglycan layer that aids the mobility of the metal ions to the cells and assists the interface between the nanoparticles and bacterial cell walls^33^. The explicit antibacterial potential of Zn-doped TiO_2_ nanoparticles is due to the combined bimetallic effect of two metals, i.e., Zinc and Titanium, which present synergism between the two metal atoms.

The synthesized pure and doped TiO_2_ nanoparticles were also tested for their inhibitory assay against four common food fungal stains, namely *B. cineria*, *P.expansum*, *C. albicans,* and *F. oxysporum*. The MIC values for pure TiO_2_ nanoparticles were 25 µg/mL against* C. albicans* and *F. oxysporum*, *B. cineria,* and *P. expansum*. The dopants also behaved differently from their bulk counterparts. The MIC values determined for Zn-doped TiO_2_ were found to be 25 µg/mL, whereas, for Cu-doped and Se-doped TiO_2_ nanoparticles, the MIC values determined were 10 µg/mL against *B. cineria*. In the case of the fungal strain, *P. expansum* MTCC 4485, the MIC values were found to be 25 µg/mL for Zn-doped TiO_2_ nanoparticles, whereas the MIC values were found to be 10 µg/mL for Cu-doped and Se-doped TiO_2_ nanoparticles. The MIC values determined against *C. albicans* MTCC 3017 were 25 µg/mL for Zn-doped TiO_2_ nanoparticles and 10 µg/mL for Cu-doped and Se-doped TiO_2_ nanoparticles. The MIC values determined against *F. oxysporum* MTCC 1755 were 25 µg/mL for Zn-doped, Cu-doped and Se-doped TiO_2_ nanoparticles. The titanium tetrachloride used as a precursor for synthesizing TiO_2_ nanoparticles has shown inhibitory results against all the tested fungal strains at each concentration. The results of the MIC assay are illustrated in Table 1 and depict that the doped TiO_2_ nanoparticles have outstanding inhibitory effects against the tested fungal strains.

**Figure 3:**
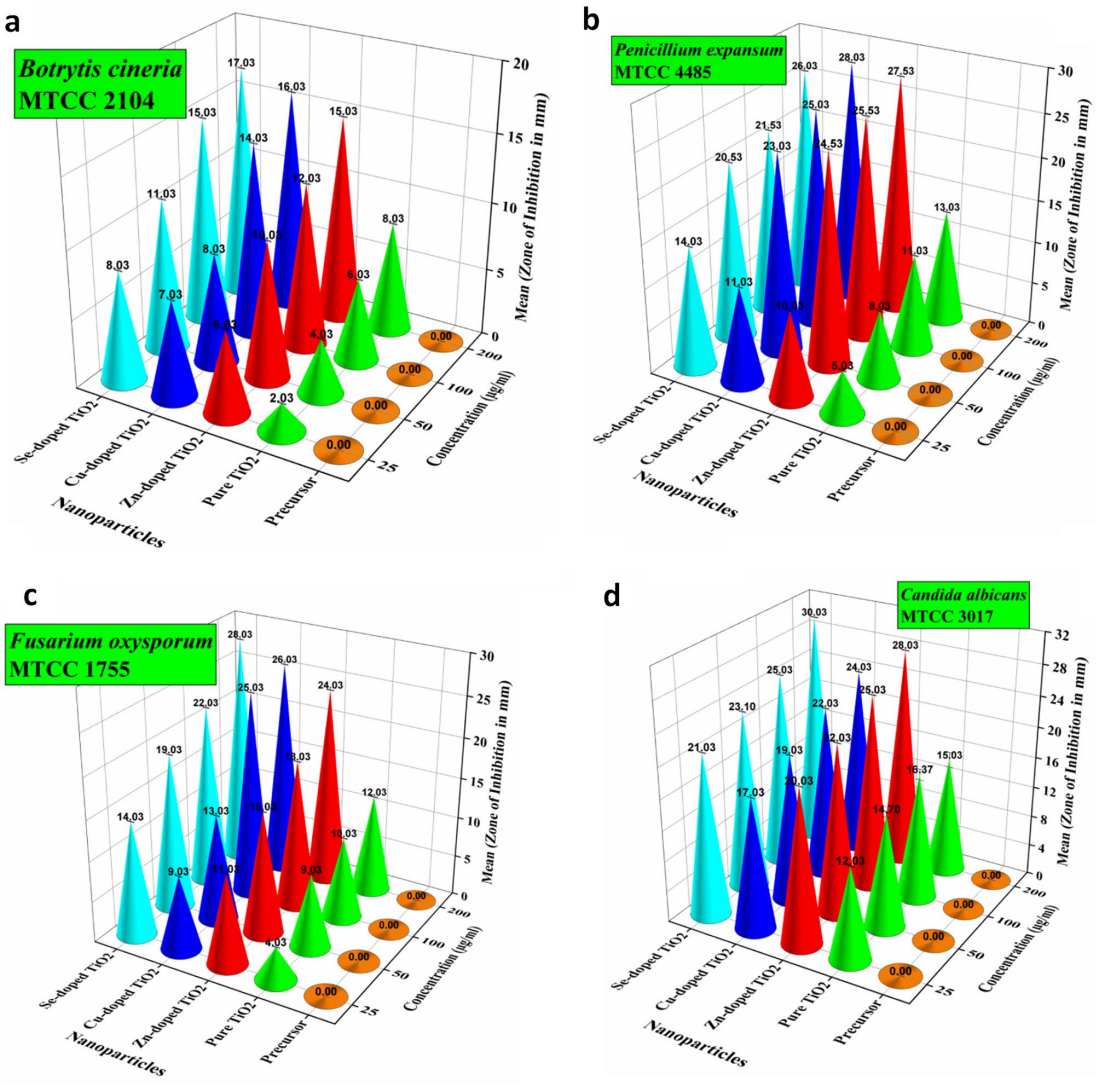
Antifungal activity of pure and doped TiO_2_ nanoparticles against (**a**) *B. cineria*; (**b**); *P. expansum*; (**c**) *F. oxysporum*; (**d**) *C. albicans*.

The antifungal efficiency of the synthesized nanoparticles was evaluated at the different concentrations of 25, 50, 100, and 200 µg/mL against four fungal strains, i.e., *B. cineria* MTCC 2014, *P. expansum* MTCC 4485, *F. oxysporum* MTCC 1755 and *C. albicans* MTCC 3017 (Fig. 3a–d). It is inferred from the antifungal potential of the synthesized nanoparticles that the maximum zone of inhibition was exhibited by Se-doped TiO_2_ nanoparticles against *C. albicans* MTCC 3017 followed by Cu-doped TiO_2_ nanoparticles against *P. expansum* MTCC 4485 and Zn-doped nanoparticles against *C. albicans* MTCC 3017*.* It has been understood from the antifungal response of the synthesized nanoparticles towards selected fungal pathogens that all of the synthesized nanoparticles are effective against the tested fungal strains. Maneerat and Hayata^34^ reported the antifungal activity of TiO_2_ nanoparticles against *P. expansum* that possess the potential for control of post-harvest diseases. Siripatrawan and Kaewklin demonstrated the potent antifungal potential of Chitosan-titanium nanoparticles against *Penicillium* sp^35^. The zinc oxide nanoparticles at a size of 70 nm inhibited the growth of *Botrytis cinerea* and *Penicillium expansum* but showed more sensitivity towards *Penicillium expansum*. The underlying mechanism study reveals that ZnO nanoparticles affected the progress of *Botrytis cinerea* by disturbing their cellular functionality, eventually distorting mycological hyphae. However, for *Penicillium expansum*, ZnO nanoparticles hindered the progression of conidia and conidiophores, which also caused the demise of the fungal hyphae^36^.

The robust antibacterial and antifungal potential of Zn-doped TiO_2_ nanoparticles amid all of the synthesized nanoparticles has prompted them to be carefully selected for further amalgamation into active packages for food packaging applications.

**Figure 4:**
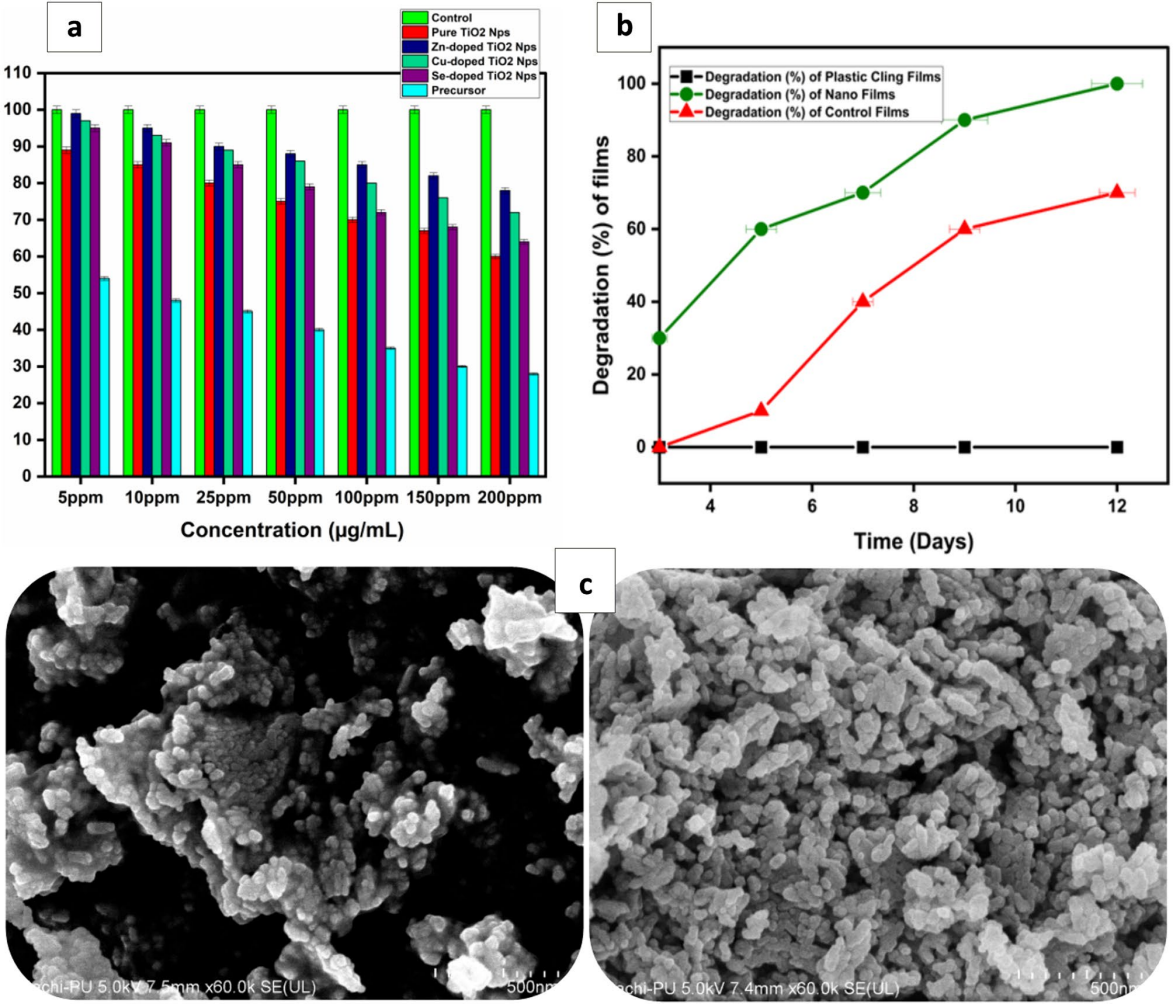
(**a**) MTT assay of the nanoparticle-treated cells and their comparison with control (without any nanoparticles treated cells); (**b**) Biodegradation of the prepared films; (**c**) FE-SEM micrographs of the synthesized nanoparticles.

The cell viability of the splenocytes after treating them with different concentrations of nanoparticles (5-200 µg/mL) has been presented in Fig. 4a. It has been observed from the cytotoxicity assay that the nanoparticles have not caused any significant change in cell viability when compared to the control. The cell viability was up to 60% at the maximum concentration, i.e., 200 µg/mL in sets getting only pure TiO_2_ nanoparticles. The cell viability was > 65% when the concentration of pure TiO_2_ nanoparticles decreased to 150 µg/mL. In another set where Zn-doped TiO_2_ nanoparticles were added at different concentrations, the cells showed the viability of > 85% from concentrations ranging between 5 and 100 µg/mL and > 78% at the maximum concentration, i.e., 200 µg/mL. In the case of Cu-doped TiO_2_, the cell viability decreased from 97 to 72% when the concentration of nanoparticles gradually increased from 5 to 200 µg/mL, respectively. Se-doped TiO_2_ nanoparticles showed 95% to 64% cell viability when concentration was elevated from 5 to 200 µg/mL, respectively. As a result, it has been determined that, compared to control groups containing no nanoparticles, the mitogenic response was much lower in cells receiving Zn-doped TiO_2_ nanoparticles than in pure TiO_2_ nanoparticles. Rajendran et al. reported 99% cell viability when zinc oxide nanorod-doped titanium dioxide nanosheets were used at 50 mg/mL concentrations^37^. The aforementioned in vitro study suggested no cytotoxicity of zinc and titanium under 50 mg/mL, which agrees with our mitogenic studies. However, due to their lower cytotoxicity, Zn-doped TiO_2_ nanoparticles were selected to be introduced into the active food packages.

The FE-SEM micrographs of Zn-doped TiO_2_ nanoparticles reveal the structural symmetry of the synthesized nanoparticles, as shown in Fig. 4c. The clusters of nano-grains fall apart; this shows their monodisperse nature with hexagonal symmetry along with aggregation. The size identified from the FE-SEM analysis is approximately 20–35 nm, harmonising with the size recognized from XRD patterns and zeta sizing analysis. The FE-SEM micrographs of Zn-doped TiO_2_ nanoparticles revealts that these nanoparticles are completely stable without forming clusters.

The results observed from the antibacterial, antifungal, and cytotoxicity response of the synthesized Zn-doped TiO_2_ nanoparticles have shown excellent potential to be merged into the active food packages in the context of safety towards humans. The food packaging films were prepared by reinforcing gelatin-pectin blends with Zn-doped TiO_2_ nanoparticles and characterized for the following physicomechanical properties:

The prepared active nano-reinforced packaging was neat, transparent, and flexible with a glossy appearance. The depth of the gelatin-pectin blends amalgamated with Zn-doped TiO_2_ nanoparticles was 0.183 mm, while for the control film, it was found to be 0.132 mm. The mean thickness of the active nano-reinforced package (0.183 mm) was significantly increased as compared to the control films (0.132 mm). This was perhaps owing to the bi-metallic joint effect of zinc and titanium interweaving amid the networks of the pectin and gelatin blends that improve the resultant anticipated properties of the film.

The primary objective of adding nanofillers to the produced films is to enhance their function. However, the evaluation of moisture content is a critical parameter to mend the functionality of the films instead of control from microbial attack. In the case of nano-reinforced packaging, the moisture content has decreased significantly to 12.8%, whereas it is 88% for control films, as revealed in Table 2. Loading nanofillers into the films' matrix greatly influenced the lowering of the moisture content of the nanopack. This is likely due to the potent linkages produced by nanoparticles in the mixtures of gelatin and pectin. Priyadarshi et al. reported a decrease in moisture content and moisture absorption values by 29% and 86–87%, respectively, when chitosan films were cross-linked with citric acid^38^. The reduced moisture content values of the nano-reinforced package perceived in this study are highly advantageous for successful food packaging substantial.

**Table 2:** Summary of the physicomechanical attributes of the prepared nano-reinforced packaging.

Physicomechanical properties of the films	Nano-reinforced packaging	Control film
Thickness (mm)	0.183 ± 0.01^a^	0.132 ± 0.01^a^
Moisture content (%)	12.8 ± 0.02^b^	88 ± 0.02^b^
Water solubility (%)	30 ± 0.01^a^	60 ± 0.02^b^
Swelling behaviors of films (%)	98 ± 0.01^a^	1030 ± 0.02^b^
Water vapour permeability (g/cm/pa/sec)*10^−10^	0.022 ± 0.01^a^	0.13 ± 0.03^c^
Tensile strength (MPa) N/mm^2^	3.27 ± 0.02^b^	0.57 ± 0.01^a^
% Elongation at break	227.99 ± 0.03^c^	241.54 ± 0.02^b^
Force required to puncture the film (N) or burst strength	91.36 ± 0.04^d^	16.12 ± 0.03^c^

Values labeled in both columns show statistical significance among the groups at *p* ≤ 0.05, with no statistical difference.

*All the data obtained were expressed as means (triplicates) ± standard error with Tukey’s hinges using Origin Pro 2022 (OriginLab Corporation) software. Statistical differences were considered at a significance level of *p* ≤ 0.05.

Water solubility and swelling have been the signs of integrity and water resistance of the composite materials^20, 39^. For control films, the water solubility has been discovered to be 60%, whereas, in the case of nano-reinforced package, it has drastically dropped to 30%, as shown in Table 2. Similar findings have been investigated for the swelling index of the films, where control films swelled up to 1030%, and in the case of nanofilms, it dropped to 98%. The hydrophilic nature of the gelatin and pectin is the primary cause of the higher water solubility and swelling index values. However, the reduced values for water solubility and water swelling for nano pack mainly result from the strong hydrogen bonds between the biopolymer matrix and the duo metallic effect of zinc and titanium that made the film’s structure compact, enhancing their sealing properties. Shahabi-Ghahfarrokhi and Babaei-Ghazvini^21^ have also reported a reduction in moisture content, absorption, and solubility in water for starch-kefiran-ZnO green nanocomposite as a food packaging application. According to Tuna et al., the solvent particles of the medium are initially engrossed by the polymers, which enter the macromolecular system, thus compelling the chains of polymers separately^40^. However, this causes the freeing of the polymer structure, which releases the films’ components into the surrounding medium after some time. Therefore, the commencement of the solubility of the film begins.

Water vapour permeability is an imperative parameter in food packaging applications to be determined to lessen moisture transmission from food to the environment, thus affecting the food quality. Hence, the WVP values of the packaging material should be as low as feasible because the moisture content of the food packaging may affect the food’s quality^41^. It is also well-recognized that the polysaccharides exhibit inadequate water vapour barrier capabilities and are highly sensitive to moisture owing to their hydrophilic character^42^. The hydrophilic nature of glycerol also greatly affects the water vapour permeability of the films. A free hydroxyl group is present in its structure that can boost the increase or decrease of sogginess, causing greater water vapour transmission^43^. The WVP of the control and nano-reinforced films are shown in Table 2. The WVP has been diminished from 0.13 × 10^−10^ to 0.022 × 10^−10^ g/cm/pa/sec with the amalgamation of nanoparticles. The control films were susceptible to moisture and exhibited deprived water vapour barrier properties due to their hydrophilic character. Additionally, the decrease in WVP may be brought on by interactions between polymers and nanoparticles, resulting in a decline in the hydrophilic nature of films and a corresponding reduction in the WVP of film samples. The results presented in Table 2 were statistically significant at *p* ≤ 0.05.

Mechanical properties of the food packaging films are imperative in resisting mechanical stress and sustaining reliability during handling, shipping, and long-term storage. Glycerol is one of the essential attributes in the film-making components which enter the polymer chain, thus lowering the inter-chain interactions and plasticization impact resulting in amplified elongation at break and reduced tensile strength of the polymer films^44, 45^. However, the mechanical attributes of the nanofilms were characterized and associated with the control film to assess the reinforcing effect of doped nanoparticles in the gelatin and pectin film blends. The control film’s tensile strength (TS) was measured at 0.57 MPa, which was in line with the findings reported by Otoni et al.^46^ for the composite palatable films from diverse sources^47^. However, in the Zn-doped TiO_2_-reinforced gelatin film, the TS has improved significantly to 3.27 MPa, as shown in Table 2. Compared to control films, a similar trend of increased % elongation at break and burst strength was detected for nano-reinforced packaging.

Contact angle measurements of the water drop placed on the film surface were evaluated to determine the hydrophobic character of the gelatin and pectin blended films. The contact angle measurements of the water droplets depict the hydrophobic nature of the nano-reinforced films as contrasted with control films. The results of the water contact angle of the films were in line with the water vapour permeability of the films. However, contact angle evaluation of the films showed that the nano-reinforced films were significantly more hydrophobic than the control films, 70° for the control film and 98° for the nano-reinforced film. This is primarily explained by the robust interaction between nanoparticles and the gelatin matrix, which forms amide and hydrogen bonds with the proteins (gelatin) and polysaccharides (pectin) chains and a reduction in the free hydrophilic groups. This increases the nano film’s surface cohesiveness and surface hydrophobicity. Hence, it can be concluded that the nano-reinforced gelatin and pectin blended film caused elevation in the hydrophobicity of the film surface.

The prepared control and nano-reinforced films were measured for their surface color and opacity. The parameters of *L* (lightness), *a* (green–red), *b* (blue-yellow), ΔE (difference between the colour of standard background and sample), YI (yellowness index), and WI (whiteness index) values are shown in Table 3. The control films were transparent without any colour, but the addition of Zn-doped TiO_2_ nanoparticles slightly affected the colour attributes of the nano-reinforced film. The lightness values of the nano-reinforced film increased slightly due to the incorporation of white-colored powdered nanoparticles, but for control films, it decreased significantly (*p* < 0.05). The incorporation of Zn-doped TiO_2_ nanoparticles has reduced redness (chromaticity parameter a-value), and yellowness (chromaticity parameter b-value), but the total colour difference (ΔE) has increased significantly (*p* < 0.05). Compared to neat control films, the yellowness and whiteness indices have also decreased for nano-reinforced films. A similar trend has also been observed by Roy and Rhim for carrageenan film incorporated with different concentrations of zinc oxide nanoparticles stabilized by melanin^48^. Opacity is an additional significant feature to be measured in the case of a food packaging film as the consumer favours more translucent films to look into the whole product. However, transparency leads to an overall more dynamic package, thus significantly impacting its advertising and consumer acceptance. Opacity was also increased from 60 to 63% on both black and white backgrounds upon nano-reinforcement of films, indicating slight variation in opacity of the prepared films. The results presented in Table 3 were statistically significant at *p* value ≤ 0.05. According to Priyadarshi et al., the transparency of crosslinked and plasticized chitosan films increased by 81% while their opacity decreased^38^.

**Table 3:** Color measurements values of the prepared films.

Film sample	L	a	b	ΔE	YI	WI	Opacity on white background (%)	Opacity on black background
Control film	80.1 ± 0.01	− 2.1 ± 0.03	12.3 ± 0.01	31.63 ± 0.01	21.93 ± 0.02	76.52 ± 0.01	60	60
Nano-reinforced film	76.2 ± 0.02	− 2.3 ± 0.03	10.7 ± 0.02	31.83 ± 0.01	20.06 ± 0.02	73.81 ± 0.03	63	63

*All the data obtained were expressed as means (triplicates) ± standard error with Tukey’s hinges using Origin Pro 2022 (OriginLab Corporation) software. Statistical differences were considered at a significance level of *p* ≤ 0.05.

The biodegradability of the control and nano-reinforced film samples was evaluated using a natural soil burial degradation test. Loss in the weight of films over time is an essential indicator for detecting degradation. The loss of weight (%) for all film samples after the soil burial study is shown in Fig. 4b. From the weight loss percentage, it is evident that control films degraded more slowly than nano-reinforced films. Gradual weight loss was observed for both types of films, as shown in Fig. 4b. During the 12th day of the experiment, it was noted that nano-reinforced films degraded completely. In contrast, for control films, on the 12th day of the experiment, average 60% weight loss of the films was observed, which implies that the control films took a longer time to be degraded than nano-reinforced films. It is clear from the experimental biodegradation tests that nano-reinforced films disintegrated into natural soil faster than control films. The fast biodegradation of nano-reinforced films is probably due to the nano charge that induces micro-cracks and other food packaging material failures that trigger its degradation over time.

The films prepared were assessed for their antibacterial and antifungal potential against common noxious food pathogens such as Gram-positive (*S. aureus* and *B. pumilus*) and Gram-negative bacterial cells (*E. coli* and *P. oleovorans*) and fungal strains such as *B. cineria*, *C. albicans, F. oxysporum, and P. expansum*, respectively. The nano-reinforced films showed a maximum inhibition zone against Gram-negative bacterial cells compared to Gram-positive cells, as shown in Table 4. The control films showed no zone of inhibition against Gram-positive and Gram-negative bacterial cells. In the case of fungal strains maximum zone of inhibition was observed against *P. expansum,* followed by *F. oxysporum*, *B. cineria,* and *C. albicans*. In contrast, control films failed to demonstrate any antibacterial or antifungal effect against either food-deteriorating bacterial or fungal pathogens tested, as shown in Table 4. The Zn-doped TiO_2_ nanoparticles in the film matrix function as lethal to food pathogens and furnish biodegradable antimicrobial nano-reinforced films for food storage and transportation.

**Table 4:** Inhibitory zone of prepared films against microbial strains.

Microbial culture	Zone of inhibition (mm)	
	Nano-reinforced packaging	Control film (w/o nanoparticles)
*Staphylococcus aureus* MTCC 737	0 ± 0.00	22 ± 0.02
*Bacillus pumilus* MTCC 1607	0 ± 0.00	18 ± 0.02
*Pseudomonas oleovorans* MTCC 617	0 ± 0.00	20 ± 0.02
*Escherichia coli* MTCC 1687	0 ± 0.00	25 ± 0.02
*Botrytis cineria* MTCC 2014	0 ± 0.00	24 ± 0.02
*Penicillium expansum* MTCC 4485	0 ± 0.00	35 ± 0.02
*Fusarium oxysporum* MTCC 1755	0 ± 0.00	30 ± 0.02
*Candida albicans* MTCC 3017	0 ± 0.00	21 ± 0.02

*All the data obtained were expressed as means (triplicates) ± standard error with Tukey’s hinges using Origin Pro 2022 (OriginLab Corporation) software. Statistical differences were considered at a significance level of *p* ≤ 0.05.

**Figure 5:**
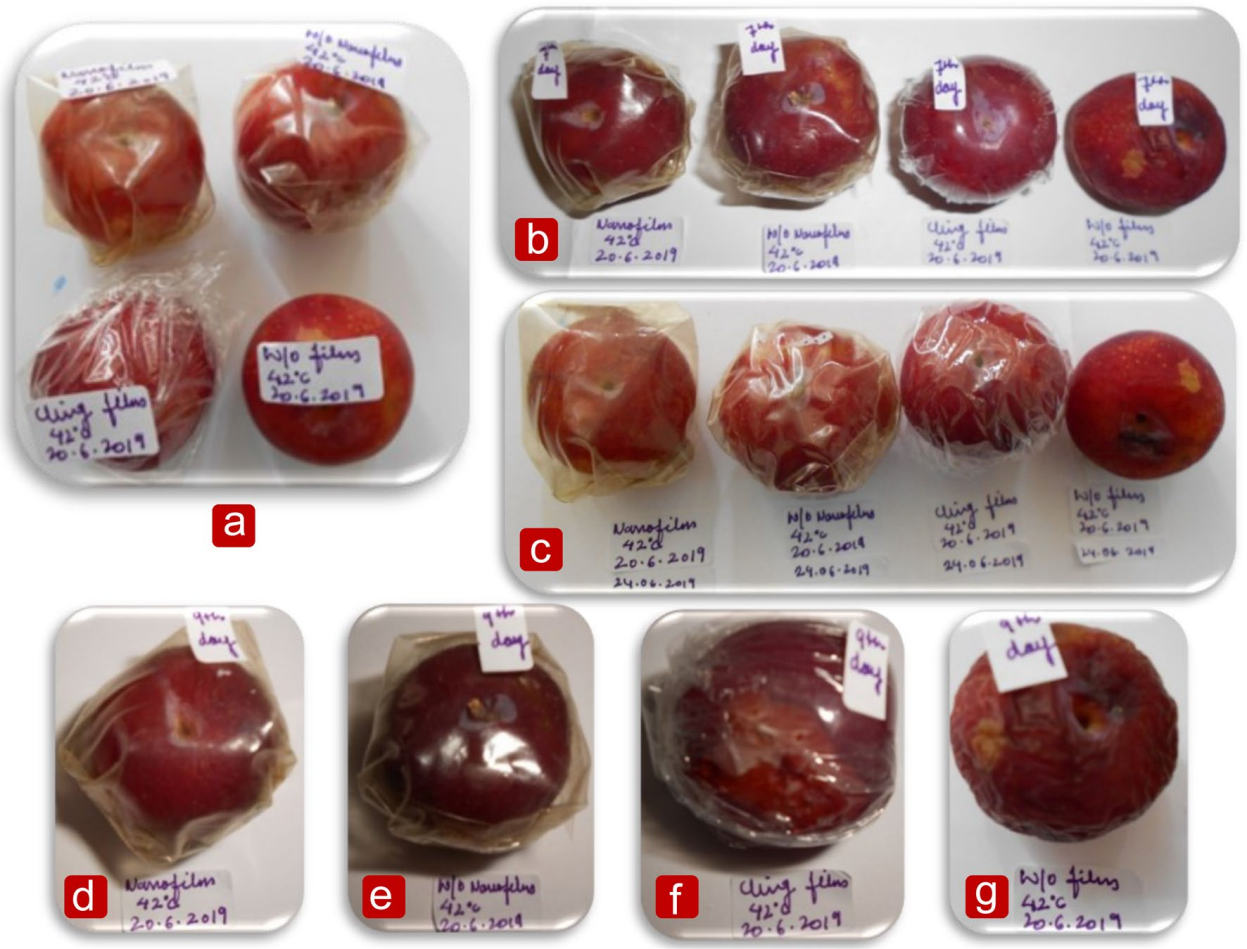
Evaluation of films for the shelf life of Plums, (**a**) Primarily wrapped plums, (**b**) Plums wrapped in different films after 4 days of storage, (**c**) Plums wrapped in different types of films after 7 days of storage, (**d**) Plums wrapped in prepared nano-reinforced package after 9 days of storage, (**e**) Plums wrapped in prepared control film (w/o nps) after 9 days of storage, (**f**) Plums wrapped in plastic cling films after 9 days of storage, (**g**) Plums without any film after 9 days of storage.

The packaging efficiency of the prepared nano-reinforced packaging was tested on plums (*Oemleria cerasiformis*). Four sets of fresh cherry plums were used: packed in nano-reinforced packaging, packed in control films (w/o nanoparticles), packed in plastic cling films, and unpacked plums. For around 16 days, these sets were stored in air at room temperature (42 ± 2 °C) on a laboratory shelf, and the physical changes that occurred during storage in each group were studied. Numerous researchers followed a similar method to assess how packaging materials affected food products' ability to maintain quality, such as bread pieces^49^, meat^50^, and fresh food^51^. To the best of our knowledge, the practical application of food packaging based on Zn-doped TiO_2_-reinforced gelatin and pectin blends has been studied for the first time, as shown in Fig. 5a–g.

After seven days of storage, plastic film-wrapped plums produced an apparent green mildew texture with numerous moldy spots, oozed sticky juice on the surface, and altered hue from purple-red to yellow, as shown in Fig. 5f, coupled with a strong rotten smell. The plums retained their quality for up to 9 days when wrapped in films made without nanoparticles, as shown in Fig. 5e, which served as positive control films. After four days of storage, the unpacked plum sample began to degrade, as seen in Fig. 5c,g. As demonstrated in Fig. 5d, which explains the proper function of the nanoparticles in the films, the plums packed with Zn-doped TiO_2_ nano-reinforced films were found fresh up to 16 days of examination without any putridity, with a smooth finish, with no seepage of the juice.

**Figure 6:**
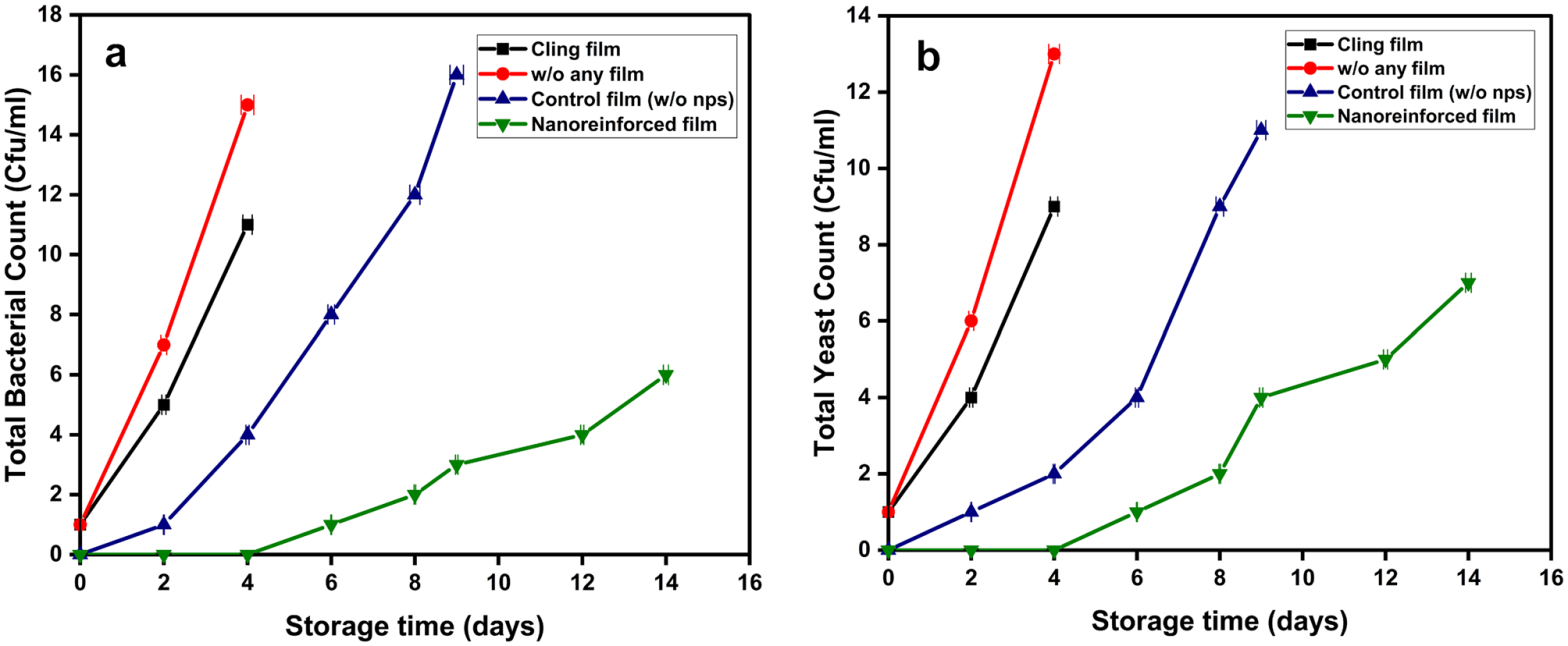
(**a**) Total bacteria count perceived for different categories of films; (**b**) Total yeast and molds count perceived for different types of films.

According to Fig. 6a,b, the total bacterial count and total yeast count for cling film, plum samples without any film, and control film (without nanoparticles) were greater than those stored in nano-reinforced films. The findings indicated that the prepared nano-reinforced packaging potentially serves as a good food packaging material that can shield the food from infectious deterioration and extend the shelf life of food stored in these food packages.

## Conclusions

TiO_2_ and ZnO nanoparticles have practical implications as potent antimicrobials with GRAS status. Pure and food-grade tailored metal (Zn, Cu, Se) doped TiO_2_ nanoparticles were successfully synthesized and characterized for their physicochemical characteristics (UV–visible spectral analysis, XRD patterns, and DLS analysis). The Zn-doped TiO_2_ nanoparticles also showed potent antimicrobial effects against Gram-positive, Gram-negative, and fungal pathogens with lower cytotoxicity response towards splenocytes. These tested nanoparticles were incorporated to prepare biodegradable nano-reinforced packaging. The biodegradable nano-reinforced packaging materials were efficaciously tested for their physicomechanical properties. These nano-reinforced packagings were used to evaluate the shelf life of plums, a perishable fruit, which showed an extension in the shelf life of up to 16 days with lower microbial load and without compromising the quality during storage. It has been concluded that the prepared active nano-reinforced packaging exhibited excellent capability to be used as a promising biodegradable food wrapper substitute for plastic components. The present study reveals that the practical implications of nanoparticles in food packaging are less detrimental than the consumption of nanoparticles as a food constituent. There is always a myth in mind related to nanomaterials that they may enter the food chain during their production, causing DNA damage, disruption of cell membranes, and demise. However, very few in vivo studies have been undertaken on the prolonged consequences of nano-foods on human health. There should be appropriate recommended tagging and followed guidelines for the promotion of nano-related products, which will help to upsurge the tolerability of nano-based products among consumers. Thus, if regulated correctly, exploitation of these nanotechnologies may perform a noteworthy part in refining food processing, superiority, and reduced food loss for the well-being of humanity, disease control, and future sustainability.

### Supplementary Information

Supplementary Tables.

## Data Availability

All available data has been presented in the paper.
