# Supplementary material for: Biodegradable nano-reinforced packaging with improved functionality to extend the freshness and longevity of Plums Oemleria cerasiformis
Source: Sci Rep. 2023 Sep 4;13:14583. doi: 10.1038/s41598-023-41640-1 (PMC10477292; doi:10.1038/s41598-023-41640-1)
Supplement: Supplementary file 1 — Supplementary Tables. [file 41598_2023_41640_MOESM1_ESM.docx]

**Biodegradable nano-reinforced packaging with improved functionality to extend the freshness and longevity** of **Plums *Oemleria cerasiformis***

Manpreet Kaur Mann^1^ and Balwinder Singh Sooch^1*^

^1^ Enzyme Biotechnology Laboratory, Department of Biotechnology, Punjabi University, Patiala-147002.

E-mail address: soochb@pbi.ac.in

Table 1 Zone of inhibition measurements (in mm) of synthesized pure and metal doped TiO_2_ nanoparticles against Gram positive bacteria (*Staphylococcus aureus* MTCC 737 and *Bacillus pumilus* MTCC 1607)

|  | ***Staphylococcus aureus* MTCC 737** | | | ***Bacillus pumilus* MTCC 1607** | |
| --- | --- | --- | --- | --- | --- |
| **Concentration (µg/ml)** | **Samples** | **Mean (Zone of Inhibition)** | **SE of mean** | **Mean (Zone of Inhibition)** | **SE of mean** |
| **µg/ml** |  | **mm** | **mm** | **mm** | **mm** |
| 25 | Titanium dioxide Salt | 0.00 | 0.00 | 0.00 | 0.00 |
| 25 | Pure TiO_2_ nanoparticles | 3.03 | 0.03 | 3.03 | 0.03 |
| 25 | Zn-doped TiO_2_ nanoparticles | 1.03 | 0.03 | 8.03 | 0.03 |
| 25 | Cu-doped TiO_2_ nanoparticles | 5.03 | 0.03 | 1.03 | 0.03 |
| 25 | Se-doped TiO_2_ nanoparticles | 2.03 | 0.03 | 1.07 | 0.06 |
| 50 | Titanium dioxide Salt | 0.00 | 0.00 | 0.00 | 0.00 |
| 50 | Pure TiO_2_ nanoparticles | 4.03 | 0.03 | 4.03 | 0.03 |
| 50 | Zn-doped TiO_2_ nanoparticles | 2.03 | 0.03 | 10.03 | 0.03 |
| 50 | Cu-doped TiO_2_ nanoparticles | 7.03 | 0.03 | 3.10 | 0.05 |
| 50 | Se-doped TiO_2_ nanoparticles | 3.03 | 0.03 | 2.07 | 0.06 |
| 100 | Titanium dioxide Salt | 0.00 | 0.00 | 0.00 | 0.00 |
| 100 | Pure TiO_2_ nanoparticles | 7.03 | 0.03 | 5.03 | 0.03 |
| 100 | Zn-doped TiO_2_ nanoparticles | 4.03 | 0.03 | 12.10 | 0.05 |
| 100 | Cu-doped TiO_2_ nanoparticles | 9.03 | 0.03 | 4.10 | 0.05 |
| 100 | Se-doped TiO_2_ nanoparticles | 4.03 | 0.03 | 3.10 | 0.05 |
| 200 | Titanium dioxide Salt | 0.00 | 0.00 | 0.00 | 0.00 |
| 200 | Pure TiO_2_ nanoparticles | 8.03 | 0.03 | 6.03 | 0.03 |
| 200 | Zn-doped TiO_2_ nanoparticles | 9.03 | 0.03 | 22.03 | 0.03 |
| 200 | Cu-doped TiO_2_ nanoparticles | 10.03 | 0.03 | 7.03 | 0.03 |
| 200 | Se-doped TiO_2_ nanoparticles | 6.03 | 0.03 | 4.03 | 0.03 |

*The diameter of zones of inhibition is expressed as the means (triplicate) ± standard error with significance level p ≤ 0.05

Table 2 Zone of inhibition measurements (in mm) of synthesized pure and metal doped TiO_2_ nanoparticles against Gram negative bacteria (*Pseudomonas oleovorans* MTCC 617 and *Escherichia coli* MTCC 1687)

|  | ***Pseudomonas oleovorans* MTCC 617** | | | ***Escherichia coli* MTCC 1687** | |
| --- | --- | --- | --- | --- | --- |
| **Concentration (µg/ml)** | **Samples** | **Mean (Zone of Inhibition)** | **SE of mean** | **Mean (Zone of Inhibition)** | **SE of mean** |
| **µg/ml** |  | **mm** | **mm** | **mm** | **mm** |
| 25 | Titanium dioxide Salt | 0.00 | 0.00 | 0.00 | 0.00 |
| 25 | Pure TiO_2_ nanoparticles | 8.03 | 0.03 | 9.03 | 0.03 |
| 25 | Zn-doped TiO_2_ nanoparticles | 12.03 | 0.03 | 11.03 | 0.03 |
| 25 | Cu-doped TiO_2_ nanoparticles | 7.03 | 0.03 | 8.03 | 0.03 |
| 25 | Se-doped TiO_2_ nanoparticles | 6.03 | 0.03 | 7.03 | 0.03 |
| 50 | Titanium dioxide Salt | 0.00 | 0.00 | 0.00 | 0.00 |
| 50 | Pure TiO_2_ nanoparticles | 9.03 | 0.03 | 13.03 | 0.03 |
| 50 | Zn-doped TiO_2_ nanoparticles | 13.03 | 0.03 | 13.03 | 0.03 |
| 50 | Cu-doped TiO_2_ nanoparticles | 9.03 | 0.03 | 10.03 | 0.03 |
| 50 | Se-doped TiO_2_ nanoparticles | 8.03 | 0.03 | 8.03 | 0.03 |
| 100 | Titanium dioxide Salt | 0.00 | 0.00 | 0.00 | 0.00 |
| 100 | Pure TiO_2_ nanoparticles | 12.03 | 0.03 | 14.03 | 0.03 |
| 100 | Zn-doped TiO_2_ nanoparticles | 14.03 | 0.03 | 16.03 | 0.03 |
| 100 | Cu-doped TiO_2_ nanoparticles | 11.03 | 0.03 | 11.03 | 0.03 |
| 100 | Se-doped TiO_2_ nanoparticles | 10.03 | 0.03 | 11.03 | 0.03 |
| 200 | Titanium dioxide Salt | 0.00 | 0.00 | 0.00 | 0.00 |
| 200 | Pure TiO_2_ nanoparticles | 14.03 | 0.03 | 16.03 | 0.03 |
| 200 | Zn-doped TiO_2_ nanoparticles | 40.03 | 0.03 | 17.03 | 0.03 |
| 200 | Cu-doped TiO_2_ nanoparticles | 13.03 | 0.03 | 13.03 | 0.03 |
| 200 | Se-doped TiO_2_ nanoparticles | 12.03 | 0.03 | 13.03 | 0.03 |

*The diameter of zones of inhibition is expressed as the means (triplicate) ± standard error with significance level p ≤ 0.05

Table 3 Zone of inhibition measurements (in mm) of synthesized pure and metal doped TiO_2_ nanoparticles against *Botrytis cineria* MTCC 2104 and *Penicillium expansum* MTCC 4485

|  | ***Botrytis cineria* MTCC 2104** | | | ***Penicillium expansum* MTCC 4485** | |
| --- | --- | --- | --- | --- | --- |
| **Concentration (µg/ml)** | **Samples** | **Mean (Zone of Inhibition)** | **SE of mean** | **Mean (Zone of Inhibition)** | **SE of mean** |
| **µg/ml** |  | **mm** | **mm** | **mm** | **mm** |
| 25 | Titanium dioxide Salt | 0.00 | 0.00 | 0.00 | 0.00 |
| 25 | Pure TiO_2_ nanoparticles | 2.03 | 0.03 | 5.03 | 0.03 |
| 25 | Zn-doped TiO_2_ nanoparticles | 6.03 | 0.03 | 10.03 | 0.03 |
| 25 | Cu-doped TiO_2_ nanoparticles | 7.03 | 0.03 | 11.03 | 0.03 |
| 25 | Se-doped TiO_2_ nanoparticles | 8.03 | 0.03 | 14.03 | 0.03 |
| 50 | Titanium dioxide Salt | 0.00 | 0.00 | 0.00 | 0.00 |
| 50 | Pure TiO_2_ nanoparticles | 4.03 | 0.03 | 8.03 | 0.03 |
| 50 | Zn-doped TiO_2_ nanoparticles | 10.03 | 0.03 | 24.53 | 0.03 |
| 50 | Cu-doped TiO_2_ nanoparticles | 8.03 | 0.03 | 23.03 | 0.03 |
| 50 | Se-doped TiO_2_ nanoparticles | 11.03 | 0.03 | 20.53 | 0.03 |
| 100 | Titanium dioxide Salt | 0.00 | 0.00 | 0.00 | 0.00 |
| 100 | Pure TiO_2_ nanoparticles | 6.03 | 0.03 | 11.03 | 0.03 |
| 100 | Zn-doped TiO_2_ nanoparticles | 12.03 | 0.03 | 25.53 | 0.03 |
| 100 | Cu-doped TiO_2_ nanoparticles | 14.03 | 0.03 | 25.03 | 0.03 |
| 100 | Se-doped TiO_2_ nanoparticles | 15.03 | 0.03 | 21.53 | 0.03 |
| 200 | Titanium dioxide Salt | 0.00 | 0.00 | 0.00 | 0.00 |
| 200 | Pure TiO_2_ nanoparticles | 8.03 | 0.03 | 13.03 | 0.03 |
| 200 | Zn-doped TiO_2_ nanoparticles | 15.03 | 0.03 | 27.53 | 0.03 |
| 200 | Cu-doped TiO_2_ nanoparticles | 16.03 | 0.03 | 28.03 | 0.03 |
| 200 | Se-doped TiO_2_ nanoparticles | 17.03 | 0.03 | 26.03 | 0.03 |

*The diameter of zones of inhibition is expressed as the means (triplicate) ± standard error with significance level p ≤ 0.05

Table 4 Zone of inhibition measurements (in mm) of synthesized pure and metal doped TiO_2_ nanoparticles against *Fusarium oxysporum* MTCC 1755 and *Candida albicans* MTCC 3017

|  | ***Fusarium oxysporum* MTCC 1755** | | | ***Candida albicans* MTCC 3017** | |
| --- | --- | --- | --- | --- | --- |
| **Concentration (µg/ml)** | **Samples** | **Mean (Zone of Inhibition)** | **SE of mean** | **Mean (Zone of Inhibition)** | **SE of mean** |
| **µg/ml** |  | **mm** | **mm** | **mm** | **mm** |
| 25 | Titanium dioxide Salt | 0.00 | 0.00 | 0.00 | 0.00 |
| 25 | Pure TiO_2_ nanoparticles | 4.03 | 0.03 | 12.03 | 0.03 |
| 25 | Zn-doped TiO_2_ nanoparticles | 11.03 | 0.03 | 20.03 | 0.03 |
| 25 | Cu-doped TiO_2_ nanoparticles | 9.03 | 0.03 | 17.03 | 0.03 |
| 25 | Se-doped TiO_2_ nanoparticles | 14.03 | 0.03 | 21.03 | 0.03 |
| 50 | Titanium dioxide Salt | 0.00 | 0.00 | 0.00 | 0 |
| 50 | Pure TiO_2_ nanoparticles | 9.03 | 0.03 | 14.70 | 0.35 |
| 50 | Zn-doped TiO_2_ nanoparticles | 15.03 | 0.03 | 22.03 | 0.03 |
| 50 | Cu-doped TiO_2_ nanoparticles | 13.03 | 0.03 | 19.03 | 0.03 |
| 50 | Se-doped TiO_2_ nanoparticles | 19.03 | 0.03 | 23.10 | 0.05 |
| 100 | Titanium dioxide Salt | 0.00 | 0.00 | 0.00 | 0.00 |
| 100 | Pure TiO_2_ nanoparticles | 10.03 | 0.03 | 16.37 | 0.68 |
| 100 | Zn-doped TiO_2_ nanoparticles | 18.03 | 0.03 | 25.03 | 0.03 |
| 100 | Cu-doped TiO_2_ nanoparticles | 25.03 | 0.03 | 22.03 | 0.03 |
| 100 | Se-doped TiO_2_ nanoparticles | 22.03 | 0.03 | 25.03 | 0.03 |
| 200 | Titanium dioxide Salt | 0.00 | 0.00 | 0.00 | 0.00 |
| 200 | Pure TiO_2_ nanoparticles | 12.03 | 0.03 | 15.03 | 0.98 |
| 200 | Zn-doped TiO_2_ nanoparticles | 24.03 | 0.03 | 28.03 | 0.03 |
| 200 | Cu-doped TiO_2_ nanoparticles | 26.03 | 0.03 | 24.03 | 0.03 |
| 200 | Se-doped TiO_2_ nanoparticles | 28.03 | 0.03 | 30.03 | 0.03 |

*The diameter of zones of inhibition is expressed as the means (triplicate) ± standard error with significance level p ≤ 0.05
